# *Tribulus terrestris* and Sport Performance: A Quantitative and Qualitative Evaluation of Its Advertisement and Availability via Online Shopping in Six Different Countries

**DOI:** 10.3390/nu16091320

**Published:** 2024-04-28

**Authors:** Juan F. Garcia, Jesús Seco-Calvo, Soledad Arribalzaga, Raquel Díez, Cristina Lopez, M. Nelida Fernandez, Juan J. Garcia, M. Jose Diez, Raul de la Puente, Matilde Sierra, Ana M. Sahagún

**Affiliations:** 1 Department of Mechanical, Informatics and Aerospatiale Engineering, University of Leon, 24071 Leon, Spain; juan.garcia.sierra@unileon.es; 2 Department of Physiotherapy, Institute of Biomedicine (IBIOMED), University of Leon, 24071 Leon, Spain; licenciada.arribalzaga@gmail.com; 3 Department of Psychology, Faculty of Medicine, Basque Country University, 48940 Leioa, Spain; 4 Department of Biomedical Sciences, Institute of Biomedicine (IBIOMED), Veterinary Faculty, University of Leon, 24071 Leon, Spain; rdielz@unileon.es (R.D.); clopcd@unileon.es (C.L.); mnferm@unileon.es (M.N.F.); jjgarv@unileon.es (J.J.G.); mjdiel@unileon.es (M.J.D.); rpueg@unileon.es (R.d.l.P.); msiev@unileon.es (M.S.); amsahp@unileon.es (A.M.S.)

**Keywords:** athlete, dietary supplement, doping, internet, sport, *Tribulus terrestris*

## Abstract

Dietary supplements are commonly used among athletes, and the Internet may be an easy source of these products. *Tribulus terrestris* is an herbal supplement with multiple properties. Of interest to athletes are reports that its consumption can lead to muscle mass gain and a faster recovery process. The objective of this cross-sectional study was to determine the availability of *Tribulus terrestris* via the Internet in six countries (Canada, Puerto Rico, Russia, Spain, Ukraine, and the United States of America) via a specifically designed computer program. The characteristics of the websites selling this substance, the country from which it can be purchased, the route of administration, and recommendations for its use were analyzed. The results of the study show that this supplement is marketed mainly in Russia, Ukraine, and Spain on many websites that are mostly dedicated to sports products. Just over half of the webpages (59.14%) identified only distribute this supplement within the same country. The main claims for its consumption refer to sports performance benefits, but there are also claims that it may improve male hormone levels and sexual function. Athletes should be encouraged to seek professional advice prior to ingesting this supplement to ensure that it is suitable for their specific training and sports objectives.

## 1. Introduction

Dietary herbal supplements have become popular among consumers, especially athletes, as a way of naturally improving endurance and strength performance or addressing their nutritional deficits. They primarily contain substances that are commonly found in the diet, such as vitamins, minerals, plants, amino acids, and other compounds or their derivatives [1,2], and are free from banned substances [3]. Nutritional status is one of the most important determinants of exercise performance [4], and dietary supplements can help provide essential nutrients, enhance weight gain, or improve athlete appearance and muscle building [5]. Nevertheless, before consuming them, it is important that athletes have knowledge about the appropriate conditions for their use, the potential benefits and adverse effects, and risks associated with their use [2], as despite the high consumption and popularity of these products, concerns regarding their efficacy and safety have been raised.

**Figure 1:**
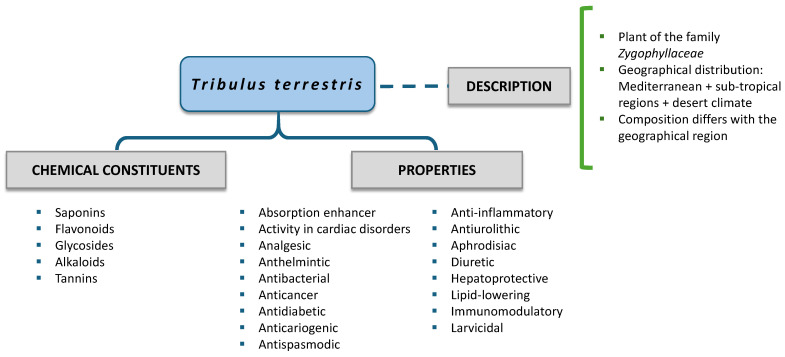
General characteristics of *Tribulus terrestris*.

Among herbal dietary supplements, *Tribulus terrestris* (TT) has become particularly popular among athletes. This plant grows in South Africa, Australia, India, and Europe, and its fruits are employed in traditional Chinese and Ayurvedic medicine in India to treat different conditions [6,7] (Figure 1). TT belongs to the Zygophyllaceae family, and many compounds with a variety of biological properties and chemical structures have been identified in its extract, including steroidal saponins, flavonoids, tannins, terpenoids, polyphenol carboxylic acids, and alkaloids [8].

The most significant bioactive components in TT are flavonoids (quercetin, kaempferol, and isorhamnetin) and steroidal saponins [9]. These phenolic compounds are naturally occurring in plants and have antioxidant properties, mainly associated with their effects on glutathione, transduction pathway signals, and reactive oxygen species [10,11]. In TT, the most common saponins are furostanol and spirostanol saponins, and more than 70 different compounds [9] with different chemical compositions according to their geographical origins have been identified [12]. Steroidal saponins are responsible for the antimicrobial and anti-inflammatory properties of TT [13]. Protodioscin is the most dominant component in TT fruits, and it is considered the main pharmacologically active steroidal saponin [14]. This plant and the compounds derived from it are claimed to have diuretic [15], aphrodisiac [16], hypoglycemic, antidiabetic [17,18], lipid-lowering [19], antioxidant [3], anti-inflammatory [4], analgesic [5], antibacterial [20], hepatoprotective [21], and anticariogenic [6] properties. 

Worldwide, there are many pharmaceutical preparations and herbal supplements containing extracts standardized in steroidal saponins. Since the early 1980s, TT extract has been an attractive unconventional medicine product used in Western countries as a testosterone booster, libido enhancer, and adaptogenic aid for healthy, physically active men. Currently, TT is mainly advertised as a testosterone booster that may improve the androgenic status of males with hypogonadism and enhance athletic performance [22]. 

Of interest to athletes, the potential ergogenic effects of TT include improvements in sports performance [23], post-exercise recovery [24], and strength, as well as the stimulation of skeletal muscle hypertrophy [25] and muscle anabolism [24,26]. This supplement may also attenuate muscle [27] and oxidative damage [28], thereby diminishing inflammation [29]. As explained above, the saponins present in TT are suggested to be the active ingredients responsible for these effects [30]. These compounds may activate testosterone and luteinizing hormone (LH) production, which, in turn, stimulates muscle growth [6,31]. For this reason, TT is also used to as an alternative to androgenic substances that are banned in sports [30]. Moreover, the polyphenols and flavonoids contained in TT have antioxidant activities through their free radical scavenging properties [6,26]. 

Supplements associated with sports performance are easily accessible for athletes, and their use has increased in recent years [1,2,32]. It is estimated that 40–100% of athletes consume dietary supplements (depending on the definition of supplement, the sport type, and the competition level) to improve their athletic performance or health, rebalance nutritional deficiencies, and/or accelerate recovery [33]. In 2020, the global trade in dietary supplements accounted for more than USD 140 billion, and an annual growth rate of 8.6% is estimated for the next decade [7]. However, inappropriate use or abuse of these products may damage health or impair athletic performance, and some supplements may be contaminated with prohibited substances [34]. In recent years, the online market for dietary supplements has undergone remarkable expansion. However, although motivations for their consumption [35,36] and/or advice given by trainers [37] or pharmacists [38,39] have been assessed, to the best of the authors’ knowledge, there has been no research on the potential acquisition of sports herbal dietary supplements through Internet sites and information provided to athletes. 

Thus, the aim of this study was to determine the availability of TT for sale through the Internet in six different countries. We documented the characteristics of webpages that sell this product, the dosage forms commonly offered, and the information about this dietary supplement provided to athletes and consumers, including information shown in the labeling of the product being advertised. Our hypothesis was that it is easy for potential consumers, including athletes, to obtain TT from the Internet through different websites and from different countries. 

## 2. Materials and Methods

This study was carried out by a multidisciplinary working group that included pharmacology, sport medicine, and cybersecurity experts. The pharmacology experts were responsible for the conceptualization of the study and the analysis of the data obtained. Information was reviewed in pairs, and potential disagreements were resolved by consensus among all members. The experts in sport medicine designed the study and interpreted the data obtained, whereas the member with expertise in cybersecurity developed the search software and programmed the search. The Internet search and data evaluation took 18 days (1–18 October 2023).

### 2.1. Design

This study was carried out to assess the online availability of TT for sale from certain countries (Canada, Puerto Rico, Russia, Spain, Ukraine, and the United States of America). Of these countries, Russia, Ukraine, and Puerto Rico were selected, as they have been monitored by the World Anti-Doping Agency (WADA) since October 2022 [40]. The United States of America (USA) was selected due to the high number of athletes in this country and the high sales of sports supplements, accounting for more than USD 1.4 billion in annual revenue [41]. Finally, Canada and Spain were selected because these countries host the Compliance Review Committee (CRC) and the International Federation of Bodybuilding and Fitness (IFBB), respectively.

A computer program adapted for this study was used, the characteristics of which are described below. A cross-sectional study was performed, with observations of the sales of this product taken at the point in time that the study was completed. For this purpose, a purchasing simulation was carried out for each of the countries listed above using the computer program designed for this study. 

### 2.2. Search Engine

To gather the information we needed from the Internet, the latest version of our web crawler was used, and this was modified to adapt to the latest Google’s SERP (Search Engine Result Page) changes, as described in another parallel study [42]. We also masked our activity using a VPN.

The crawler was used to automatically visit a list of predefined websites (seeds) and extract and/or analyze the information contained on these websites. The keywords utilized, “*Tribulus terrestris*” and “buy”, were translated into the languages spoken in each of the countries included: Latin (for Spanish- and English-speaking countries), Russian, and Ukrainian. The crawler obtained the list of URLs to visit directly from the search engine’s result page, that is, Google’s result page (SERP).

The major limitations were the same as those encountered in our previous research: mandatory continuous scrolling through the SERP’s configuration and the omission of related results.

To avoid mandatory continuous scrolling, we used Selenium 4.20, a browsing automation software tool, to simulate a user scrolling down to the bottom of the SERP until no more results were loaded. We also purged related results (which now only showed after the end of the page was reached and were highly irrelevant or duplicated) and limited the seeds to just one per domain. 

As for the VPN, we used Urban VPN (free), as our previous research showed it to be the best among both paid and free alternatives. Since it does not offer data for Puerto Rico, we obtained those results using Google’s region option, which still offers reliable and useful results.

### 2.3. Data from the Webpages Analyzed

Although the program searched for websites selling TT, the webpages found were checked by the research group, as some of them were focused on other purposes (e.g., books). The characteristics of the websites analyzed included the number of pages that sold TT and the website type (grouped as sports, supermarkets, pharmacy or parapharmacy, nutritional supplements and similar, laboratories, stores (selling different types of goods), or others outside these categories). Indications for TT consumption were also evaluated, as well as if the product offered was for oral, parenteral, or another route of administration. Finally, the potential purchase of other compounds using the same websites was also checked. For this reason, advertising for the sale of 3 doping substances (dehydroepiandrosterone, also known as DHEA, oxandrolone, and androstenedione) was assessed.

### 2.4. Statistical Analysis

Data were collected and stored in Microsoft Excel 2016, and the statistical analysis was carried out using SPSS version 24. The results are expressed as frequencies and percentages. A Chi-squared test was performed to compare the characteristics of the websites found. A value of *p* ≤ 0.05 was taken as the level of significance.

## 3. Results

Table 1 summarizes the characteristics of the websites reviewed. TT was mentioned on a total of 1024 webpages. Russia (22.17%) and Spain (19.14%) were the countries with the most webpages advertising this product. After reviewing the websites that offered this compound for purchase, these two countries were also associated with the most webpages (21.38% each). 

The statistical analysis revealed significant differences between countries regarding the percentage of webpages actually selling TT (*p* < 0.05), with Spain, Puerto Rico, and the USA containing the most websites. Similar proportions were found for countries in the following groupings: Canada, Russia, and Ukraine in one grouping and Spain, Puerto Rico, and the USA in the other grouping. 

Webpages offering this product were mostly dedicated to sports (39.17%), particularly those from Russia and Ukraine (73.55% and 72.41%, respectively). For the USA and Puerto Rico, it was found that TT can be purchased mainly from webpages advertising dietary products (61.84% and 31.29%, respectively). Spain showed an intermediate situation between these two possibilities (webpages focused on sports or dietary supplements), and in Canada, the types of websites were much more diversified. On the other hand, webpages related to parapharmacy appeared to be nearly negligible.

A total of 58.90% of the websites only shipped orders locally within their country. Similar to the abovementioned results, this was particularly the case for webpages from Russia and Ukraine (78.71% and 96.55%, respectively), whereas for webpages from the USA, sales were mainly made worldwide (44.74%). 

Likewise, 24.02% of the webpages offered compounds other than TT. Of the websites that also sold the three doping substances that we searched for, the vast majority had DHEA available, whereas androstenedione and oxandrolone were offered at lower rates. Significant differences between countries were found regarding the supply of products other than TT (*p* < 0.05).

The characteristics of the TT products offered on the websites are presented in Table 2. Regarding the route of administration, practically all websites sold TT as an oral commercial formulation, and there were only a few cases where seeds of the plant were offered. 

Most of the websites (68.70%) marketed this compound based on its association with improvements in sports performance, with the percentage of websites promoting this feature ranging from 61.46% in Canada to 76.12% in Russia. The other major recommendation of this product (44.60% of webpages) was as a supplement to increase male hormone levels and sexual function (18.42% of websites from the USA to 70.96% of those from Russia). It should also be noted that some websites found in Spain, Russia, and Ukraine did not include any information regarding the commercial products they offered.

## 4. Discussion

The present study was carried out to analyze the online sale of TT in different countries to evaluate the ability to acquire this commonly used herbal dietary supplement. The information contained on the labels of the products offered for sale was also assessed, allowing us to determine how these products were provided and advertised in terms of their forms of administration and recommendations for use. 

The key results of this study are that TT is commonly available through the Internet in the six countries investigated, primarily from sports webpages (39.17%). The information provided regarding the benefits of its consumption include improvements in sports performance (68.70% of webpages), without further specifications on how such improvements can be achieved, and increases in male hormone levels and sexual function (44.60%). In some cases, no information about the product was given. 

According to Qureshi et al. [26], who surveyed users older than 30 years of age, the consumption of this product is mainly motivated by a desire to gain muscle mass. TT is frequently consumed by bodybuilders and athletes because it can increase testosterone concentrations and thereby promote muscle growth, at least according to the information provided on its label [26,43]. This is in line with our findings, as many webpages emphasize this advantage for male athletes. Its physiological effects occur due to increases in the anabolic and androgenic actions of testosterone [44]. Improvements in certain biomarkers in active adults have been shown [23], but research on the potential effects on muscle damage and anti-inflammatory and hormonal biomarkers is lacking [45]. From these results, it is clear that TT products are mainly indicated for oral administration under different dosage forms or as seeds.

Despite its advertised properties, some studies have not been able to demonstrate the ability of TT to modify body composition [25,30,46], strength [25], or anaerobic performance [30]. However, a tendency to improve the recovery process by increasing serum testosterone and maintaining a favorable testosterone/cortisol ratio, which results in less fatigue, has been shown [30]. Several studies [38,39] have pointed out that pharmacists may be a source of information in the selection and purchase of these products. However, our study only took into account the types of websites that sell TT. 

Currently, TT is mainly advertised as being able to enhance sports performance and improve androgenic status in cases of male hypogonadism, as it is considered a testosterone booster [8]. A previous study suggested that TT extracts are usually recommended as a way to improve sexual desire and athletic performance [8], either as a single-ingredient product or as a component of nutritional supplements. 

Despite this evidence regarding the intake of dietary products such as TT, the intake of these products is often controversial. Advertisements for these products contain health claims that, in some cases, can lead to misinformation about the supposed benefits to be obtained by consuming them [32]. These health claims try to establish a relationship between the consumption of a substance and the improvement in a certain physical condition [47]. However, often, there are no evidence-based studies to support these claims. Regarding TT, although a certain dose is recommended on the label, the most appropriate dosage for athletic performance has not yet been established. A previous study showed that in resistance-trained males, a dosage of 3.2 mg/kg daily does not enhance exercise performance [48]. However, it is unclear whether a higher dose would produce positive effects.

In spite of its wide consumption, TT supplementation is not considered risk-free, and kidney and liver damage has been described [49,50]. In addition, in some instances, some of these products may be contaminated with substances that are not declared on the label and are sometimes prohibited by the WADA [51]. Therefore, the Australian Institute of Sport (AIS) classifies TT within category D of the AIS Sports Supplement Framework [52] and does not recommend its use. The most common contaminants are anabolic androgenic steroids (such as testosterone and nandrolone) and ephedrine [53,54,55]. Contamination can be caused by a poor manufacturing process (cross-contamination) or deliberate adulteration by the manufacturer (intentional contamination) [51]. The former is associated with a low concentration of prohormones due to the production of multiple supplements and/or poor cleaning of machinery [56]. In the case of intentional contamination, prohormones are added to the supplement in order to enhance its effect [56]. In any case, the involuntary ingestion of prohibited substances is not an excuse for athletes who fail a doping control, as they are responsible for everything they ingest, and therefore have to face the corresponding sanction if they test positive for a banned substance [56]. 

Helle et al. [57] observed that doping substances were found in 23% of dietary supplements sold through a Dutch website. In another study [32], 12–58% of products tested were contaminated, which suggests that unintentional doping could occur due to the omission of some banned substances on the labels. This information becomes even more relevant as 3–4.5% of supplements [32,58] were found to contain compounds in amounts that could result in serious adverse effects, such as hepatotoxicity, cardiac and hormonal problems, and even carcinogenesis [59]. In addition to these health complications, other negative consequences should be considered, such as the loss of sponsors, the damage to an athlete’s image, and potential financial penalties [58]. Thus, the risk-to-benefit ratio should be carefully considered by athletes before taking these supplements.

Moreover, we observed that the same webpages offering TT sometimes advertise compounds banned by the WADA, such as DHEA and, much less frequently, oxandrolone and androstenedione, despite the consequences that their consumption may have for athletes. The case of DHEA can also be misleading because, despite being included in the WADA list of prohibited substances [60], it is possible to purchase it as a dietary supplement, at least in the USA. This is of particular interest to athletes, as some of them tend to take more than one supplement simultaneously. Moreover, the use of dietary supplements may favor doping use [61,62]. It is worth mentioning that these doping substances are marketed for their anabolic properties (oxandrolone, DHEA, and androstenedione) and for being a precursor of testosterone (DHEA and androstenedione) [63]. The main bioactive component of TT, protodioscin, has been reported to possibly increase the level of dehydroepiandrosterone [64]. Thus, it would not be unusual for both products (TT and DHEA) to be purchased on the same website for consumption.

Dietary supplements are regulated as foods and, unlike drugs, they are not assessed for their efficacy and safety. National legislations are also more permissive when acquiring these products inside vs. outside one’s country. We note that with the exception of Ukraine and Russia, probably due to the current war situation, TT products are routinely sold worldwide or at least to some other countries from the other four countries assessed in our study. 

Athletes should be well informed about which dietary supplements are allowed and how to select them, e.g., brands, modes of administration, and timing of ingestion [38]. They should also be informed about potential contamination with banned substances as, even if the consumption of these substances does not extend to a sanction by the WADA [60], it may cause serious adverse effects to their health [41]. For this reason, certified doping-free supplements should always be recommended [65]. 

Regarding TT, comprehensive knowledge about the effects of this supplement should be provided by suppliers, thereby helping customers to choose a suitable product and avoid the unintentional intake of banned substances.

## 5. Conclusions

Based on the results of this study, it is concluded that TT is globally offered and easily purchased from a substantial number of webpages. Most websites where TT is offered are sports websites, followed by stores. The main claims regarding its consumption refer to sports performance benefits through increases in muscle mass, strength, and endurance, as well as a way of improving male hormone levels and sexual function. TT is mostly purchased from the country in which it is sold, but it is possible to buy it from other countries. This suggests that many of the purchases take place in athletes’ home countries, although there is also an important global market, or at least a market extending to several countries.

Consumers, in particular athletes, should be encouraged to take advice from health professionals (sports physicians and/or nutritionists) about which supplements are suitable for their type of training and sports objectives, as well as brands that are certified, in order to prevent potential adverse events and avoid the unintentional intake of doping substances through contamination.

## Figures and Tables

**Table 1:** Characteristics of the websites offering *Tribulus terrestris* (TT) from the countries assessed in this study.

		Canada	Puerto Rico	Russia	Spain	Ukraine	USA
General characteristics	No. of webpages reviewed	179	186	227	196	144	92
	Does not connect *	22 ^a^	8 ^c^	42 ^a^	5 ^b,c^	28 ^a^	0 ^b^
	Does not sell TT	48	38	31	36	26	16
	Sells TT *	109 ^a^	140 ^b^	155 ^a^	155 ^b^	90 ^a^	76 ^b^
	Also sells other products *	27 ^a^	40 ^c^	84 ^c^	20 ^a^	48 ^b^	27 ^b^
	Oxandrolone	1	4	2	3	3	2
	DHEA	27	38	82	20	48	26
	Androstenedione				1	1	1
							
Types of webpages selling TT	Sports	27	26	114	49	63	5
	Supermarket	3	2		1		
	Pharmacy	7	27	2	32	2	2
	Parapharmacy	1	5	7	13	2	
	Dietary supplements	5	55	23	41	11	47
	Laboratory			2	3		1
	Store	27	25	7	16	9	21
	Other	39					
							
Shipping countries	Only ships within the country	35	85	122	71	84	30
	Worldwide	43	35	9	25	3	34
	Several countries	28	20	4	59		12
	Unknown	3		20			
							

* Significant differences (*p* < 0.05). Each superscript letter denotes a subset whose proportions do not differ significantly from each other (*p* < 0.05).

**Table 2:** Characteristics of the products containing TT offered on the websites located in the countries assessed in this study.

		Canada	Puerto Rico	Russia	Spain	Ukraine	USA
Route of administration	Oral	106	140	155	152	90	76
	Parenteral						
	Other *	3			3		
							
Recommended use of the product	1	67	79	118	85	78	69
	2	37	47	110	49	65	14
	3	3	10	16	2	17	3
	4	22	10	23	10	24	1
	5	11	6	12	2	13	6
	6	15	8	4	2		1
	No description			12	10	12	

* TT offered as seeds. 1—sports: TT advertised as a product that stimulates the growth of muscle tissue, accelerates recovery after exercise, and increases strength and endurance; 2—TT advertised as a product that increases male hormone levels and promotes sexual activity; 3—TT advertised as a product that accelerates fat burning and has hypoglycemic properties; 4—TT advertised as a product for the improvement of physical and mental stress; 5—TT advertised as a product for the improvement of renal and cardiovascular functions; 6—TT advertised as a product with anti-inflammatory and immunostimulant properties.

## Data Availability

Data are available from the corresponding author upon reasonable request.

## References

[1] Zheleva-Dimitrova D., Obreshkova D., Nedialkov P. (2012). Antioxidant activity of *Tribulus terrestris*—A natural product in infertility therapy. Int. J. Pharm. Pharm. Sci..

[2] Sellami M., Slimeni O., Pokrywka A., Kuvačić G., D Hayes L., Milic M., Padulo J. (2018). Herbal medicine for sports: A review. J. Int. Soc. Sports Nutr..

[3] Wardenaar F.C., Hoogervorst D. (2022). How sports health professionals perceive and prescribe nutritional supplements to olympic and non-olympic sthletes. Int. J. Environ. Res. Public Health.

[4] Nejati M., Dehghan P., Khani M., Sarbakhsh P. (2022). The effect of *Tribulus terrestris* supplementation on inflammation, oxidative stress, and performance of recreational runners: Study protocol for a randomized placebo-controlled trial. Trials.

[5] Heidari M.R., Mehrabani M., Pardakhty A., Khazaeli P., Zahedi M.J., Yakhchali M., Vahedian M. (2007). The analgesic effect of *Tribulus terrestris* extract and comparison of gastric ulcerogenicity of the extract with indomethacine in animal experiments. Ann. N. Y. Acad. Sci..

[6] Chhatre S., Nesari T., Kanchan D., Somani G., Sathaye S. (2014). Phytopharmacological overview of *Tribulus terrestris*. Pharmacogn. Rev..

[7] Yanala S.R., Sathyanarayana D., Kannan K. (2016). A recent phytochemical review—Fruits of *Tribulus terrestris* linn. J. Pharm. Sci. Res..

[8] Ștefănescu R., Tero-Vescan A., Negroiu A., Aurică E., Vari C.-E. (2020). A comprehensive review of the phytochemical, pharmacological, and toxicological properties of *Tribulus terrestris* L.. Biomolecules.

[9] Zhu W., Du Y., Meng H., Dong Y., Li L. (2017). A review of traditional pharmacological uses, phytochemistry, and pharmacological activities of *Tribulus terrestris*. Chem. Cent. J..

[10] Hammoda H.M., Ghazy N.M., Harraz F.M., Radwan M.M., ElSohly M.A., Abdallah I.I. (2013). Chemical constituents from *Tribulus terrestris* and screening of their antioxidant activity. Phytochemistry.

[11] Xu D., Hu M.-J., Wang Y.-Q., Cui Y.-L. (2019). Antioxidant Activities of Quercetin and Its Complexes for Medicinal Application. Molecules.

[12] Lazarova I., Ivanova A., Mechkarova P., Peev D., Valyovska N. (2011). Intraspecific variability of biologically active compounds of different populations of *Tribulus terrestris* L. (Zygophyllaceae) in South Bulgaria. Biotechnol. Biotechnol. Equip..

[13] Lombardo B., Izzo V., Terracciano D., Ranieri A., Mazzaccara C., Fimiani F., Cesaro A., Gentile L., Leggiero E., Pero R. (2020). Laboratory medicine: Health evaluation in elite athletes. Clin. Chem. Lab. Med..

[14] Dinchev D., Janda B., Evstatieva L., Oleszek W., Aslani M.R., Kostova I. (2008). Distribution of steroidal saponins in *Tribulus terrestris* from different geographical regions. Phytochemistry.

[15] Al-Ali M., Wahbi S., Twaij H., Al-Badr A. (2003). *Tribulus terrestris*: Preliminary study of its diuretic and contractile effects and comparison with Zea mays. J. Ethnopharmacol..

[16] Santos C.A., Reis L.O., Destro-Saade R., Luiza-Reis A., Fregonesi A. (2014). *Tribulus terrestris* versus placebo en el tratamiento de la disfunción eréctil: Un estudio aleatorizado, prospectivo y doble ciego. Actas Urológicas Españolas.

[17] Ercan P., El S.N. (2016). Inhibitory effects of chickpea and *Tribulus terrestris* on lipase, α-amylase and α-glucosidase. Food Chem..

[18] Samani N.B., Jokar A., Soveid M., Heydari M., Mosavat S.H. (2016). Efficacy of the hydroalcoholic extract of *Tribulus terrestris* on the serum glucose and lipid profile of women with diabetes mellitus. J. Evid. Based Complement. Altern. Med..

[19] Khan S., Kabir H., Jalees F., Asif M., Naquvi K.J. (2011). Antihyperlipidemic potential of fruits of *Tribulus terrestris* Linn. Int. J. Biomed. Res..

[20] Batoei S., Mahboubi M., Yari R. (2016). Antibacterial activity of *Tribulus terrestris* methanol extract against clinical isolates of Escherichia coli. Herba Pol..

[21] Kavitha P., Ramesh R., Bupesh G., Stalin A., Subramanian P. (2011). Hepatoprotective activity of *Tribulus terrestris* extract against acetaminophen-induced toxicity in a freshwater fish (*Oreochromis mossambicus*). Vitr. Cell. Dev. Biol.—Anim..

[22] Pokrywka A., Obmiński Z., Malczewska-Lenczowska J., Fijatek Z., Turek-Lepa E., Grucza R. (2014). Insights into supplements with *Tribulus terrestris* used by athletes. J. Hum. Kinet..

[23] Ma Y., Guo Z., Wang X. (2017). *Tribulus terrestris* extracts alleviate muscle damage and promote anaerobic performance of trained male boxers and its mechanisms: Roles of androgen, IGF-1, and IGF binding protein-3. J. Sport Health Sci..

[24] Yin L., Wang Q., Wang X., Song L.-N. (2016). Effects of *Tribulus terrestris* saponins on exercise performance in overtraining rats and the underlying mechanisms. Can. J. Physiol. Pharmacol..

[25] Rogerson S., Riches C.J., Jennings C., Weatherby R.P., Meir R.A., Marshall-Gradisnik S.M. (2007). The effect of five weeks of *Tribulus terrestris* supplementation on muscle strength and body composition during preseason training in elite rugby league players. J. Strength Cond. Res..

[26] Qureshi A., Naughton D.P., Petroczi A. (2014). A systematic review on the herbal extract *Tribulus terrestris* and the roots of its putative aphrodisiac and performance enhancing effect. J. Diet. Suppl..

[27] Talemi M.N.P.E., Ardakani S.M.P., Roozbeh B. (2021). *Tribulus terrestris* may decrease muscle damage markers following a high-intensity resistance exercise: A pilot study. Int. J. Vitam. Nutr. Res..

[28] Alzahrani S., Ezzat W., Elshaer R.E., Abd El-Lateef A.S., Mohammad H.M.F., Elkazaz A.Y., Toraih E., Zaitone S.A. (2018). Standarized *Tribulus terrestris* extract protects against rotenone-induced oxidative damage and nigral dopamine neuronal loss in mice. J. Physiol. Pharmacol. An Off. J. Polish Physiol. Soc..

[29] Heidari S., Babor T.F., De Castro P., Tort S., Curno M. (2016). Sex and gender equity in Research: Rationale for the SAGER guidelines and recommended use. Res. Integr. Peer Rev..

[30] Fernández-Lázaro D., Mielgo-Ayuso J., del Valle Soto M., Adams D.P., González-Bernal J.J., Seco-Calvo J. (2021). The effects of 6 weeks of *Tribulus terrestris* L. supplementation on body composition, hormonal response, perceived exertion, and crossFit^®^ performance: A randomized, single-blind, placebo-controlled study. Nutrients.

[31] Williams M. (2006). Dietary supplements and sports performance: Herbals. J. Int. Soc. Sports Nutr..

[32] Martínez-Sanz J., Sospedra I., Ortiz C., Baladía E., Gil-Izquierdo A., Ortiz-Moncada R. (2017). Intended or unintended doping? A review of the presence of doping substances in dietary supplements used in sports. Nutrients.

[33] Garthe I., Maughan R.J. (2018). Athletes and supplements: Prevalence and perspectives. Int. J. Sport Nutr. Exerc. Metab..

[34] Baltazar-Martins G., de Souza D.B., Aguilar-Navarro M., Muñoz-Guerra J., Plata M.d.M., Del Coso J. (2019). Prevalence and patterns of dietary supplement use in elite Spanish athletes. J. Int. Soc. Sports Nutr..

[35] Petróczi A. (2007). Attitudes and doping: A structural equation analysis of the relationship between athletes’ attitudes, sport orientation and doping behaviour. Subst. Abuse Treat. Prev. Policy.

[36] Morente-Sánchez J., Zabala M. (2013). Doping in sport: A review of elite athletes’ attitudes, beliefs, and knowledge. Sport. Med..

[37] Zmuda Palka M., Bigosińska M., Siwek M., Angelova-Igova B., Mucha D.K. (2023). Doping in sport—Attitudes of physical trainers students regarding the use of prohibited substances increasing performance. Int. J. Environ. Res. Public Health.

[38] Malek S., Taylor J., Mansell K. (2014). A questionnaire examining attitudes of collegiate athletes toward doping and pharmacists as information providers. Can. Pharm. J. Rev. Pharm. Can..

[39] Howard M.S., DiDonato K.L., Janovick D.L., Schroeder M.N., Powers M.F., Azzi A.G., Lengel A.J. (2018). Perspectives of athletes and pharmacists on pharmacist-provided sports supplement counseling: An exploratory study. J. Am. Pharm. Assoc..

[40] (2022). World Anti-Doping Agency WADA Compliance Review Committee Discusses RUSADA, Ukraine and Other Keymatters of Compliance with the World Anti-Doping Code. https://www.wada-ama.org/en/news/wada-compliance-review-committee-discusses-rusada-ukraine-and-other-key-matters-compliance.

[41] Badawy M.T., Sobeh M., Xiao J., Farag M.A. (2021). Androstenedione (a natural steroid and a drug supplement): A comprehensive review of its consumption, metabolism, health effects, and toxicity with sex differences. Molecules.

[42] Garcia J.F., Seco-Calvo J., Arribalzaga S., Díez R., Lopez C., Fernandez M.N., Garcia J.J., Diez M.J., de la Puente R., Sierra M. (2023). Online information and availability of three doping substances (anabolic agents) in sports: Role of pharmacies. Front. Pharmacol..

[43] Gupta C., Prakash D., Gupta S. (2016). Nutraceuticals for athletes. Adv. Food Technol. Nutr. Sci.—Open J..

[44] Saudan C., Baume N., Emery C., Strahm E., Saugy M. (2008). Short term impact of *Tribulus terrestris* intake on doping control analysis of endogenous steroids. Forensic Sci. Int..

[45] Fernández-Lázaro D., Fernandez-Lazaro C., Seco-Calvo J., Garrosa E., Adams D., Mielgo-Ayuso J. (2022). Effects of *Tribulus terrestris* L. on sport and health biomarkers in physically active adult males: A systematic review. Int. J. Environ. Res. Public Health.

[46] Brown G.A., Vukovich M.D., Reifenrath T.A., Uhl N.L., Parsons K.A., Sharp R.L., King D.S. (2000). Effects of Anabolic Precursors on serum testosterone concentrations and adaptations to resistance training in young men. Int. J. Sport Nutr. Exerc. Metab..

[47] Food & Drug Administration Label Claims for Conventional Foods and Dietary Supplements. https://www.fda.gov/food/food-labeling-nutrition/label-claims-conventional-foods-and-dietary-supplements.

[48] Antonio J., Uelmen J., Rodriguez R., Earnest C. (2000). The effects of *Tribulus terrestris* on body composition and exercise performance in resistance-trained males. Int. J. Sport Nutr. Exerc. Metab..

[49] Talasaz A.H., Abbasi M.-R., Abkhiz S., Dashti-Khavidaki S. (2010). *Tribulus terrestris*-induced severe nephrotoxicity in a young healthy male. Nephrol. Dial. Transplant..

[50] Ryan M., Lazar I., Nadasdy G.M., Nadasdy T., Satoskar A.A. (2015). Acute kidney injury and hyperbilirubinemia in a young male after ingestion of *Tribulus terrestris*. Clin. Nephrol..

[51] Maughan R. (2005). Contamination of dietary supplements and positive drug tests in sport. J. Sports Sci..

[52] Australian Sports Commision Supplements Benefits and Risks of Using Supplements and Sports Foods. https://www.ais.gov.au/nutrition/supplements.

[53] Aqai P., Cevik E., Gerssen A., Haasnoot W., Nielen M.W.F. (2013). High-throughput bioaffinity mass spectrometry for screening and identification of designer anabolic steroids in dietary supplements. Anal. Chem..

[54] Cavalcanti G.d.A., Leal F.D., Garrido B.C., Padilha M.C., de Aquino Neto F.R. (2013). Detection of designer steroid methylstenbolone in “nutritional supplement” using gas chromatography and tandem mass spectrometry: Elucidation of its urinary metabolites. Steroids.

[55] Judkins C., Prock P. (2012). Supplements and inadvertent doping—How big is the risk to athletes. Med. Sport Sci..

[56] Kozhuharov V.R., Ivanov K., Ivanova S. (2022). Dietary supplements as source of unintentional doping. Biomed Res. Int..

[57] Helle C., Sommer A.K., Syversen P.V., Lauritzen F. (2019). Doping substances in dietary supplements. Tidsskr. Den Nor. legeforening.

[58] Duiven E., van Loon L.J.C., Spruijt L., Koert W., de Hon O.M. (2021). Undeclared doping substances are highly prevalent in commercial sports nutrition supplements. J. Sport. Sci. Med..

[59] Maughan R.J. (2013). Quality assurance issues in the use of dietary supplements, with special reference to protein supplements. J. Nutr..

[60] (2023). World Anti-Doping Agency International Standard Prohibited List 2024. https://www.wada-ama.org/sites/default/files/2023-09/2024list_en_final_22_september_2023.pdf.

[61] Tsorbatzoudis H., Barkoukis V., Lazuras L. (2011). Determinants of Intentions for Doping in Sports in Youth: Empirical Study and Prevention Intervention in Adolescent Athletes (DIDIS-Youth). https://www.wada-ama.org/en/resources/social-science-research/determinants-intentions-doping-sports-youth-empirical-study-and.

[62] Hurst P., Schiphof-Godart L., Kavussanu M., Barkoukis V., Petróczi A., Ring C. (2023). Are dietary supplement users more likely to dope than non-users?: A systematic review and meta-analysis. Int. J. Drug Policy.

[63] Jenkinson D.M., Harbert A.J. (2008). Supplements and sports. Am. Fam. Physician.

[64] Adimoelja A., Adaikan P. (1997). Protodioscin from herbal plant *Tribulus terrestris* L. improves male sexual functions possibly via DHEA. Int. J. Impot. Res.

[65] Fernández-Lázaro D., Seco-Calvo J., Pascual-Fernández J., Domínguez-Ortega C., Del Valle Soto M., Mielgo-Ayuso J. (2022). 6-Week supplementation with *Tribulus terrestris* L. to trained male crossFit^®^ athletes on muscle, inflammation, and antioxidant biomarkers: A randomized, single-blind, placebo-controlled trial. Int. J. Environ. Res. Public Health.

